# A marvelous new glassfrog (Centrolenidae, Hyalinobatrachium) from Amazonian Ecuador

**DOI:** 10.3897/zookeys.673.12108

**Published:** 2017-05-12

**Authors:** Juan M. Guayasamin, Diego F. Cisneros-Heredia, Ross J. Maynard, Ryan L. Lynch, Jaime Culebras, Paul S. Hamilton

**Affiliations:** 1 Universidad San Francisco de Quito USFQ, ﻿Colegio de Ciencias Biológicas y Ambientales COCIBA, ﻿Instituto BIÓSFERA-USFQ, ﻿Laboratorio de Biología Evolutiva, ﻿Campus Cumbayá, ﻿Casilla Postal 17-1200-841, ﻿Quito 170901, ﻿Ecuador; 2 Universidad San Francisco de Quito USFQ, ﻿Colegio de Ciencias Biológicas y Ambientales, ﻿Instituto de Zoología Terrestre, ﻿Museo de Zoología, ﻿Quito 1709071, ﻿Ecuador; 3 The Biodiversity Group, ﻿Tucson, ﻿Arizona, ﻿USA; 4 Third Millennium Alliance, ﻿Winnetka, ﻿Illinois, ﻿USA; 5 Centro de Investigación de la Biodiversidad y Cambio Climático, ﻿Ingeniería en Biodiversidad y Recursos Genéticos, ﻿Facultad de Ciencias de Medio Ambiente, ﻿Universidad Tecnológica Indoamérica, ﻿Calle Machala y Sabanilla, ﻿Quito, ﻿Ecuador; 6 Department of Biology, ﻿Colorado State University, ﻿Fort Collins, ﻿CO, ﻿USA; 7 King’s College London, ﻿Department of Geography, ﻿London, ﻿UK; 8 Museo Ecuatoriano de Ciencias Naturales, ﻿División de Herpetología, ﻿Instituto Nacional de Biodiversidad, ﻿Quito, ﻿Ecuador; 9 Fundación Vida Silvestre Ecuador, ﻿Quito, ﻿Ecuador

**Keywords:** Amazonia, Amphibia, Centrolenidae, *Hyalinobatrachium*, Ecuador, new species, Amazonia, Amphibia, Centrolenidae, *Hyalinobatrachium*, Ecuador, nueva especie

## Abstract

*Hyalinobatrachium* is a behaviorally and morphologically conserved genus of Neotropical anurans, with several pending taxonomic problems. Using morphology, vocalizations, and DNA, a new species from the Amazonian lowlands of Ecuador is described and illustrated. The new species, *Hyalinobatrachium yaku* **sp. n.**, is differentiated from all other congenerics by having small, middorsal, dark green spots on the head and dorsum, a transparent pericardium, and a tonal call that lasts 0.27–0.4 s, with a dominant frequency of 5219.3–5329.6 Hz. Also, a mitochondrial phylogeny for the genus is presented that contains the new species, which is inferred as sister to *H. pellucidum*. Conservation threats to *H. yaku* **sp. n.** include habitat destruction and/or pollution mainly because of oil and mining activities.

## Abstract (es)

*Hyalinobatrachium* es un género de ranas Neotropicales con una morfología y comportamiento sumamente conservados, y con varios problemas taxonómicos no resueltos. Utilizando datos morfológicos, cantos y ADN, en el presente trabajo describimos una nueva especie de las tierras bajas de la Amazonía del Ecuador. La nueva especie, *Hyalinobatrachium yaku* **sp. n.**, se diferencia de todos sus congenéricos por tener una serie de puntos mediodorsales color verde oscuros en la cabeza y cuerpo, pericardio transparente, y un canto tonal con una duración de 0.27–0.4 s, con una frecuencia dominante 5219.3–5329.6 Hz. También presentamos una filogenia mitocondrial del género, la cual incluye la nueva especie y a su especie hermana, *H. pellucidum*. Las amenazas de conservación para *H. yaku* **sp. n.** incluyen principalmente la destrucción y/o contaminación del hábitat debido a actividades mineras y petroleras.

## Introduction

Among Neotropical frogs, the genus *Hyalinobatrachium* Ruiz-Carranza & Lynch, 1991 is one of the most distinguishable because of its morphological and behavioral traits. All species in this genus have a completely transparent ventral peritoneum, which means that organs are fully visible in ventral view. The reproductive behavior is also unusual, with males calling from the underside of leaves and providing parental care to egg clutches (Ruiz-Carranza and Lynch 1991, Cisneros-Heredia and McDiarmid 2007; Guayasamin et al. 2009; Delia et al. 2010).

Species identification within *Hyalinobatrachium* is complex because species tend to have a conserved morphology (Castroviejo-Fisher et al. 2011a, b), possibly related to their similar ecological constraints. Moreover, preserved specimens in the genus lose many of the color features that, in life, allow species identification. As a consequence, taxonomic discoveries usually require multiple sets of data, with vocalizations, DNA sequences, and accurate color descriptions being particularly revealing. Herein, we describe a new species of *Hyalinobatrachium* from the Amazonian lowlands of Ecuador; the new species is closely related to *H. pellucidum* (Lynch & Duellman, 1973), but is differentiated, mainly, by having a longer call and small, dark green spots on its head.

## Material and methods

*Species concept*. Species are considered as segments of separately evolving metapopulation lineages, following the conceptual framework developed by Simpson (1951, 1961), Wiley (1978), and de Queiroz (2007).

*Morphological data*. Diagnosis and description follow Lynch and Duellman (1973) and Cisneros-Heredia and McDiarmid (2007). Webbing formula follows Savage and Heyer (1967), as modified by Guayasamin et al. (2006). Taxonomy follows the proposal by Guayasamin et al. (2009). We compared *Hyalinobatrachium* specimens housed at the following collections: Instituto de Ciencia Naturales, Universidad Nacional de Colombia, Bogotá, Colombia (ICN), University of Kansas, Museum of Natural History, Division of Herpetology, Lawrence, Kansas 66045, USA (KU), Museo de Historia Natural Gustavo Orcés, Escuela Politécnica Nacional, Quito, Ecuador (MEPN), Museo de Zoología, Universidad Tecnológica Indoamérica, Quito, Ecuador (MZUTI), Museo de Zoología, Pontificia Universidad Católica del Ecuador, Quito, Ecuador (QCAZ), National Museum of Natural History, Smithsonian Institution, Washington, D.C., USA (USNM), and Museo de Zoología, Universidad San Francisco de Quito, Quito, Ecuador (ZSFQ). Morphological measurements were taken with a digital caliper to the nearest 0.1 mm, as described in Guayasamin and Bonaccorso (2004). Sexual maturity was determined by the presence of vocal slits in males and convoluted oviducts in females.

*Bioacoustics*. Sound recordings were made with a TASCAM DR-05 Portable Digital Recorder. The calls were recorded in WAV format with a sampling rate of 44.1 kHz/second with 16 bits/sample. Measurements of acoustic variables were obtained as described in Hutter and Guayasamin (2012) and Dautel et al. (2011). Notes were divided into two classes—“pulsed” and “tonal”—based upon distinct waveforms on the rendered oscillogram. Pulsed (also termed peaked) notes are defined as having one or more clear amplitude peaks and amplitude modulation (i.e., visible increases and decreases in amplitude on the oscillogram throughout the call). In contrast, tonal notes are defined as having no clear amplitude peak. A call is defined as the sound produced in a single exhalation of air. Call data from Peruvian populations of *Hyalinobatrachium pellucidum* were obtained from Wen et al. (2012), recorded from individuals found in a stream (06°25'16.7"S, 76°17'28.5"W; 523 m a.s.l.) near San José, Departamento San Martín, Peru (Wen et al. 2012).

*Fieldwork*. The new species was found in three localities in the Amazonian lowlands of Ecuador: Timburi-Cocha Research Station, near San José de Payamino (0.4819°S, 77.2842°W, 294 m; province of Orellana); near Ahuano (1.0632°S, 77.5265°W, 360 m; province of Napo), and at the Kichwa community of Kallana (1.4696°S, 77.2783°W, 325 m; province of Pastaza). Records from San José de Payamino were collected during the following sampling periods: 30 May–09 June 2012 (11 investigators, 2 teams/night); 12–19 June 2012 (12 investigators, 2 teams/night); 03–11 June 2013 (11 investigators, 2 teams/night); 16–24 June (5 investigators, 1 team); 03 July–09 August 2013 (2 investigators, 1 team). Visual encounter surveys were conducted along transects of various lengths within primary forest, secondary and riparian forest, and along streams of various sizes during each sample period except for the last, where two people surveyed 20-m diameter plots within secondary forest for 30 minutes each (Maynard et al. 2016; RJM and PSH, unpubl. data). All surveys were conducted between 19:00–00:30 h. The record from the Arajuno River is from a small stream within primary forest, obtained during fieldwork on 3–6 April, 1998 (5 researchers, surveys along stream conducted between 19:00–23:00 h). The third locality of the new species comes from a stream affluent of the Kallana River, obtained during fieldwork on 15 April, 2016 (2 investigators, surveys along stream conducted between 20:30–22:00 h).

*Evolutionary relationships*. We generated mitochondrial sequences (12S, 16S, ND1) for two individuals of the new species of *Hyalinobatrachium*. Extraction, amplification, and sequencing protocols are as described in Guayasamin et al. (2008). The obtained sequences were compared with those of all other available species of *Hyalinobatrachium*, downloaded from GenBank (https://www.ncbi.nlm.nih.gov/genbank/) and generated mostly by Guayasamin et al. (2008), Castroviejo-Fisher et al. (2014), and Twomey et al. (2014). Sequences were aligned using MAFFT v.7 (Multiple Alignment Program for Amino Acid or Nucleotide Sequences: http://mafft.cbrc.jp/alignment/software/), with the Q-INS-i strategy. MacClade 4.07 (Maddison and Maddison 2005) was used to visualize the alignment (no modifications were necessary). Phylogenetic analyses were performed under the ML criteria in GARLI 2.01 (Genetic Algorithm for Rapid Likelihood Inference; Zwickl 2006) for each mitochondrial gene and the concatenated sequences. GARLI uses a genetic algorithm that finds the tree topology, branch lengths, and model parameters that maximize lnL simultaneously (Zwickl 2006). Individual solutions were selected after 10,000 generations with no significant improvement in likelihood, with the significant topological improvement level set at 0.01; then, the final solution was selected when the total improvement in likelihood score was lower than 0.05, compared to the last solution obtained. Default values were used for other GARLI settings, as per recommendations of the developer (Zwickl 2006). Bootstrap support was assessed via 1000 pseudoreplicates under the same settings used in tree search.

### Nomenclatural acts

The electronic edition of this article conforms to the requirements of the amended International Code of Zoological Nomenclature, and hence the new names contained herein are available under that Code from the electronic edition of this article. This published work and the nomenclatural acts it contains have been registered in ZooBank, the online registration system for the ICZN. The ZooBank LSIDs (Life Science Identifiers) can be resolved and the associated information viewed through any standard web browser by appending the LSID to the prefix “http://zoobank.org/”. The LSID for this publication is: urn:lsid:zoobank.org:pub:F1221C2E-4243-4D4F-900C-21F5C2251F8B. The electronic edition of this work was published in a journal with an ISSN, and has been archived and is available from the following digital repositories: PubMed Central, LOCKSS.

## Results

### Systematics

#### Hyalinobatrachium yaku sp. n.

Taxon classificationAnimaliaAnuraCentrolenidae

http://zoobank.org/93A045E0-130D-4217-B20F-60CB55510B06

##### Holotype.

**Figure 1.**
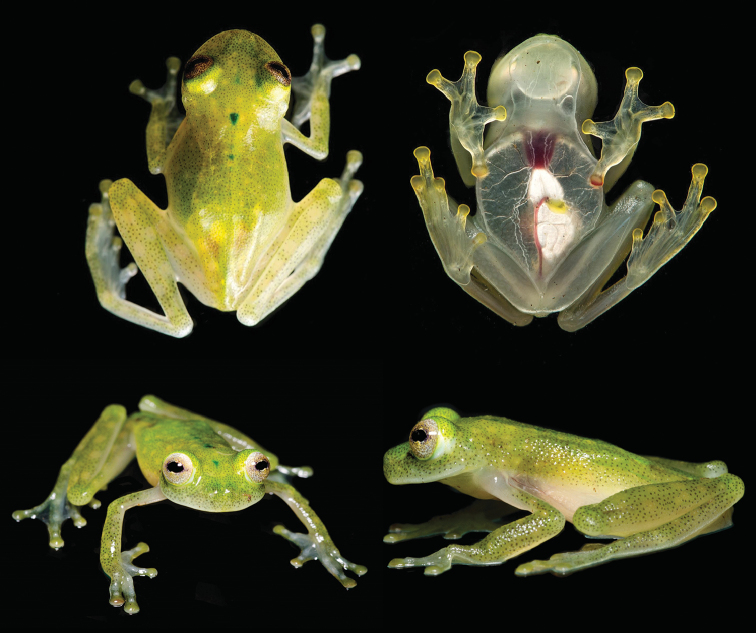
*Hyalinobatrachium yaku* sp. n. in life. Top row: adult male, MZUTI 5001, holotype, in dorsal and ventral view. Bottom row: adult male, paratype, QCAZ 55628.

MZUTI 5001 (Fig. 1), adult male collected from a stream affluent of the Kallana river (1.4696°S, 77.2784°W, 325 m), nearby the Kichwa community of Kallana, province of Pastaza, Ecuador, collected by JC and Carlos Morochz on 15 April 2016.

##### Paratopotype.

MZUTI 5002, adult male, same locality and collection data as holotype.

##### Paratypes.

QCAZ 55628 (Fig. 1), adult male, QCAZ 53352, immature male, and QCAZ 53354, 56664, juveniles, all from Timburi-Cocha Research Station (0.4800°S, 77.2829°W, 300 m) near San José de Payamino, province of Orellana, Ecuador, collected by RJM, PSH, and RLL on June 2012. ZSFQ 02322, adult female from Ahuano (1.0632°S, 77.5265°W, 360 m), province of Napo, Ecuador, collected by DFCH and Jean-Marc Touzet on 5 April 1998.

##### Generic placement.

**Figure 2.**
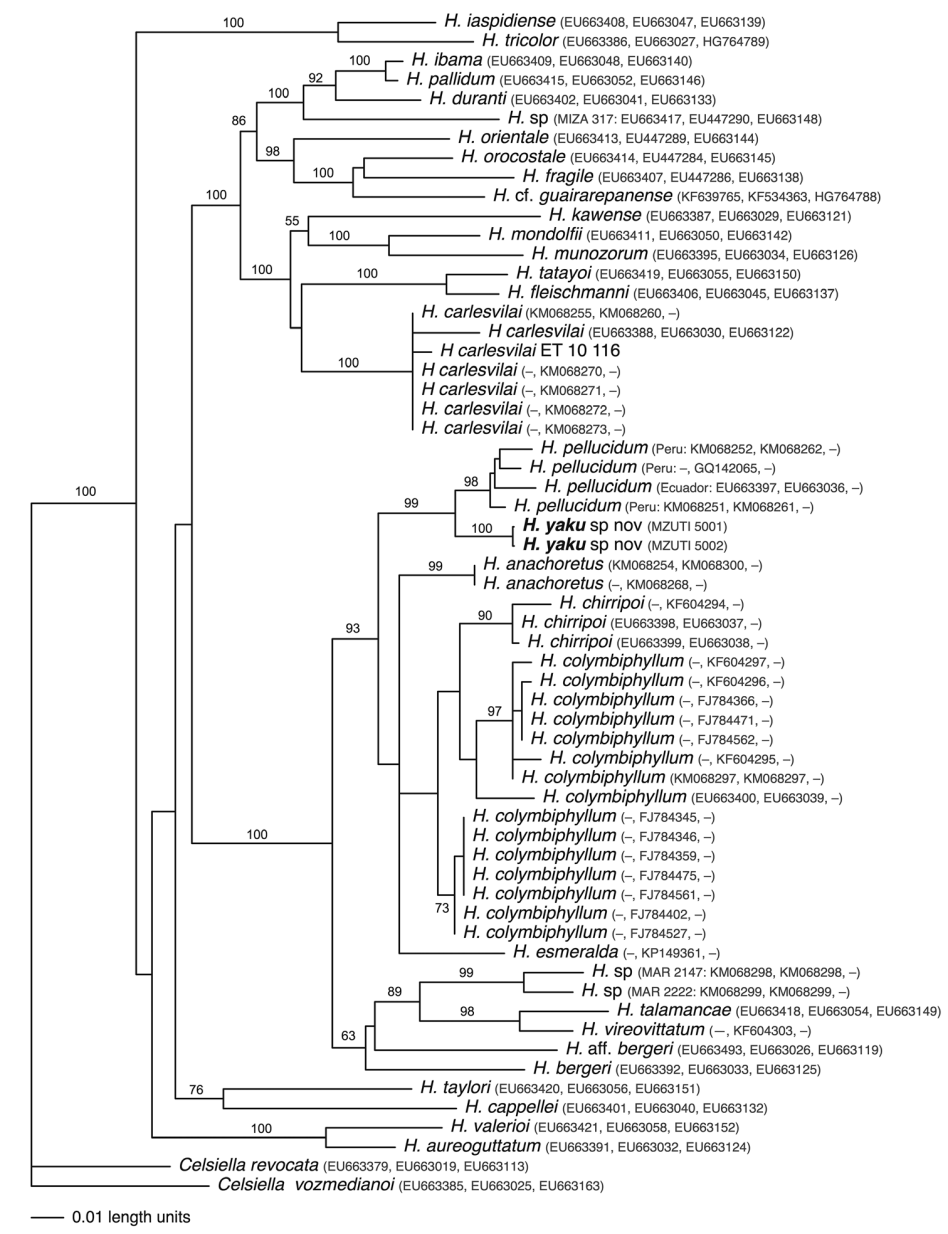
Phylogenetic relationships of *Hyalinobatrachium* inferred from combined mitochondrial genes (12S, 16S, ND1) under ML criterion. All sequences were downloaded from GenBank, except for those of the new species (Genbank codes: MF002063–68). Genbank codes cited next to species names are in the following order: 12S, 16S, ND1. Associated locality data is available at Genbank, as well as in Guayasamin et al. (2008), Castroviejo-Fisher et al. (2014), and Twomey et al. (2014).

The new species is placed in the genus *Hyalinobatrachium* (Ruiz-Carranza & Lynch, 1991, as modified by Guayasamin et al. 2009) on the basis of morphological and molecular data. The main diagnostic phenotypic traits of *Hyalinobatrachium* are: (1) ventral parietal peritoneum completely transparent; (2) digestive tract and bulbous liver covered by iridophores; (3) humeral spines absent; (4) white bones in life; and (5) males call from the undersides of leaves. All the aforementioned characteristics are shared by the new species. Additionally, analyses of three mitochondrial genes place the new species as a close relative of other *Hyalinobatrachium* species (Fig. 2); thus, generic placement in *Hyalinobatrachium* is unambiguous.

##### Diagnosis.

The following combination of characters can distinguish *Hyalinobatrachium yaku* from other glassfrogs: (1) dentigerous process of the vomer lacking teeth; (2) snout truncate in dorsal and lateral views; (3) lower half of tympanic annulus visible; tympanic membrane clearly differentiated and with coloration similar to that of surrounding skin; (4) dorsal skin shagreen; (5) ventral skin areolate; cloacal area glandular, with one tubercular slightly enameled patch on each side of the cloaca, paired round tubercles below vent absent; (6) parietal peritoneum, pericardium, kidneys and urinary bladder transparent (lacking iridophores); hepatic, gastrointestinal, and testicular peritonea covered by iridophores; (7) liver bulbous; (8) humeral spines absent; (9) basal webbing between Fingers I and II, moderate webbing between external fingers; hand webbing formula: I 2 — 2 II 0^+^ — 3^+^ III 2- — (1–2-) IV; (10) foot webbing moderate; webbing formula: I (1–1^+^) — (2–2-) II (0^+^–1) — (2^+^–2^1/3^) III 1 — 2^1/3^ IV 2^1/3^ — (1–1^1/3^) V; (11) fingers and toes with thin lateral fringes; ulnar and tarsal folds present, but low and difficult to distinguish, with thin layer of iridophores that extends to ventrolateral edge of Finger IV and Toe V; (12) nuptial excrescence present as a small pad on Finger I (Type V), prepollex not enlarged; prepollical spine not projecting (spine not exposed); (13) when appressed, finger I longer than II; (14) diameter of eye 2.1 times wider than disc on Finger III; (15) coloration in life: dorsal surfaces apple green to yellowish green with small yellow spots and minute gray to black melanophores; posterior head and anterior half of the body with few small, well-defined dark green spots placed middorsally; bones white; (16) coloration in preservative: dorsal surfaces pale cream with minute lavender to black melanophores; (17) iris coloration in life: silver to yellow, with minute dark spots that are concentrated around pupil, giving impression of a diffuse ring; (18) melanophores present on Finger IV and Toes IV–V, absent on other fingers and toes; in life, hands and feet are cream with a light green hue, with tips of fingers and toes being yellowish green; (19) males call from the undersides of leaves; advertisement call consisting of a single tonal note; call duration note 0.27–0.4 s, dominant frequency 5219–5330 Hz, with no frequency modulation; (20) males attend egg clutches located on the underside of leaves overhanging streams, clutch size unknown; (21) SVL in adult males 20.8–22.3 mm (*n* = 3), in adult female 21.1 mm (*n* = 1); (22) enameled glands absent from sides of head.

##### Comparisons with similar species.

**Figure 3.**
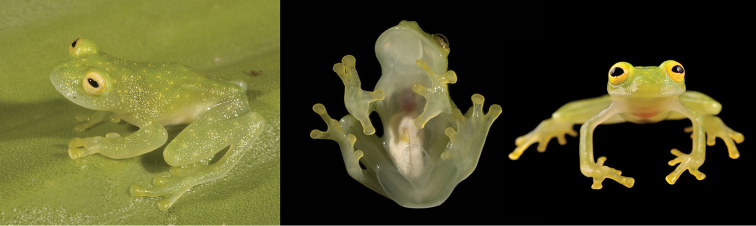
*Hyalinobatrachium pellucidum* in life. Left and center: QCAZ 4200. Right: QCAZ 41648. Photos by L. A. Coloma.

Many species of *Hyalinobatrachium* are difficult to diagnose using only morphological or chromatic characters (Castroviejo-Fisher et al. 2009; 2011); however the new species is diagnosable in life due to the presence of two unusual coloration traits: (i) the presence of middorsal dark green spots on the anterior half of the body (Fig. 1), and (ii) a completely exposed heart (parietal peritoneum and pericardium transparent). Only two other glassfrog species share, to some degree, these traits, the Central American *H. talamancae* and *H. vireovittatum*. However, phylogenetically, the new species is not closely related to *H. talamancae* nor *H. vireovittatum*. Also, the new species is easily distinguished by having a row of dark green middorsal spots (continuous middorsal line in *H. talamancae* and *H. vireovittatum*). Furthermore, they have a very disjunct distribution (*H. talamancae* and *H. vireovittatum* are found in Central America, whereas *H. yaku* inhabits the Amazonian lowlands). No Amazonian glassfrog has a dorsal pattern similar to the new species. *Hyalinobatrachium munozorum* and *H. ruedai* are sympatric with *H. yaku*, but they are distinguished by having white or mostly white pericardium (transparent in *H. yaku*), dorsal melanophores as punctuations of different sizes (uniform-sized in *H. yaku*), snout rounded in lateral view (truncate in *H. yaku*) and by lacking the row of dark green middorsal spots of *H. yaku*. *Hyalinobatrachium anachoretus* is morphologically similar to *H. yaku* but differs by lacking the midddorsal dark green spots, and by its call with a lower dominant frequency (4670–4800 Hz versus 5219.3–5329.6 in *H. yaku*). The most closely related species to *H. yaku* is *H. pellucidum* (Fig. 3); the two species differ by their call (Table 1) and dorsal color pattern (middorsal dark green spots present in *H. yaku* and absent in *H. pellucidum*; Figs 1, 3).

**Table 1.** Comparison of relevant variables of the advertisement call of *Hyalinobatrachium yaku* sp. n. and two populations of *H. pellucidum*. Time is in seconds and frequency in Hertz.

Species, museum number, source	Number of individuals/Numbers of calls	Call structure	# notes	Call duration (s)	Dominant frequency (hz)	Lower frequency (hz)	Upper frequency (hz)	Other frequencies (hz)
*H. yaku*, ﻿MZUTI 5001, this study	1/10	Tonal	1	0.27–0.4 (0.3 ± 0.03)	5219.3–5329.6 (5283.8 ± 35.0)	5207.3–5314.8 (5264.6 ± 34.6)	5236.5–5340.5 (5299.1 ± 34.1)	No
*H. pellucidum*, MEPN 14706, this study	1/6	Tonal	1	0.17–0.21 (0.18 ± 0.02)	5549.9–5667.9 (5608.4 ± 42.8)	5484.3–5575.1 (5539.4 ± 40.2)	5607.5–5691.1 (5649.5 ± 29.0)	11148.7–11303.3 (11218.9 ± 60.2)
*H. pellucidum*, ﻿MNCN 45393, uncollected individual, Wen et al. (2012)		Tonal	1	0.12–0.18 (0.15 ± 0.01)	4863.54–5408.68 Hz (5038.82 ± 190.15)	4533.0–5144.0 (4757.90 ± 191.24)	5112.0–5623.0 Hz (5284.48 ± 156.85)	No

##### Description of the holotype.

Adult male (MZUTI 5001) with SVL 20.8 mm. Head just wider than body; head width 37% of SVL; head width 1.07 times head length; head relatively short (Head length = 34% of SVL). Snout truncate in dorsal and lateral views. Loreal region slightly concave, nostrils slightly protuberant, elliptical; internarial region concave anterodorsally; canthus rostralis not well defined. Eyes small (eye diameter 12% of SVL), directed anterolaterally, eyes about 45° relative to midline. Tympanum with conspicuous dorsal inclination. Posterior half of tympanic annulus visible; tympanic membrane differentiated, pigmented as surrounding skin. Dentigerous processes on vomers lacking teeth, choanae large, circular; tongue oval, white in preservative, anterior 3/4 attached to mouth; vocal slits present, extending along floor of mouth lateral to tongue; enameled glands absent on sides of head. Ulnar fold present, but low and with very thin layer of iridophores. Relative length of fingers: I < II < IV < III; finger discs rounded, wider than toe discs; disc on Finger III 48% of eye diameter; basal finger webbing between Fingers I and II, moderate webbing between external fingers; hand webbing formula I 2 — 2 II 0^+^ — 3^+^ III 2- — 2- IV. Prepollex concealed; subarticular tubercles round, low; supernumerary tubercles absent, palmar tubercle round and small, thenar tubercle ovoid; nuptial excrescences present as a small pad on proximomedial edge of Finger I (Type V). Hind limbs slender, tibia length 59% of SVL; tarsal fold present, but low and with very thin layer of iridophores enameled; discs of toes small, round, inner metatarsal tubercle oval, small; outer metatarsal round, but very difficult to distinguish. Foot webbing moderate; webbing formula: I 1^+^ — 2 II 1 — 2^+^ III 1 — 2^1/3^ IV 2^1/3^ — 1^1/3^ V. In preservative, dorsal skin peppered with small dark melanophores; dorsal skin shagreen; skin on venter areolate; cloacal opening at level of upper thighs, cloacal ornamentation present as an enameled cloacal fold and small tubercles covered with thin layer of iridophores. Parietal peritoneum and pericardium transparent, urinary bladder lacking iridophores, liver and viscera covered by iridophores; liver bulbous.

##### Coloration in life.

In adults, dorsum apple green to yellowish green with small yellow spots and minute gray to black melanophores; posterior head and anterior half of the body with few small, well-defined dark green spots placed middorsally; the anterior-most spot generally being the largest. Hands and feet are cream with a light green hue, with tips of fingers and toes being yellowish green; melanophores absent from fingers and toes, except Finger IV and Toes IV and V. Ventrally, parietal peritoneum and pericardium transparent, with red heart fully visible; visceral peritoneum of gall bladder and urinary bladder transparent; hepatic and visceral peritonea white. Ventral vein red. Iris silver to yellow, with minute dark spots that encircle the pupil, giving the impression of diffuse rings. Bones white.

##### Coloration in preservative.

Dorsal surfaces cream dotted with minute dark lavender to black melanophores; venter uniform white; peritonea as in life. Iris white with lavender melanophores that become more numerous near the pupil. There are no traces of the characteristic middorsal dark green spots in preserved specimens.

##### Measurements.

Measurements of the type series are shown in Table 2.

**Table 2.** Meristic variation of *Hyalinobatrachium yaku* sp. n. (in mm).

Character	MZUTI 5001 (holotype)	MZUTI 5002	QCAZ 55628	ZUSF 02322
Sex	male	male	male	female
SVL	20.8	21.2	22.3	21.1
Femur	11.9	11.7	11.3	12.3
Tibia	12.3	11.7	12.5	12.4
Foot	9.6	9.9	10.0	8.9
Head length	7.1	6.6	7.3	6.7
Head width	7.6	7.4	8.1	7.7
IOD	2.3	2.2	2.3	2.4
Upper eyelid	1.9	1.7	1.6	1.3
Internarinal distance	1.6	1.5	1.7	1.6
Eye diameter	2.5	2.3	2.4	1.6
Eye-to-snout distance	3.2	3.1	3.1	2.6
Tympanum diameter	1.0	0.9	0.9	0.9
Radioulna	4.3	4.4	4.4	4.3
Hand length	5.5	6.0	6.4	4.8
Finger I	4.3	4.4	4.0	3.9
Finger II	3.8	3.9	3.7	3.5
Disc Finger III	1.2	1.1	1.1	1.1
Disc Toe IV	1.0	0.9	0.9	0.9

##### Variation.

**Figure 4.**
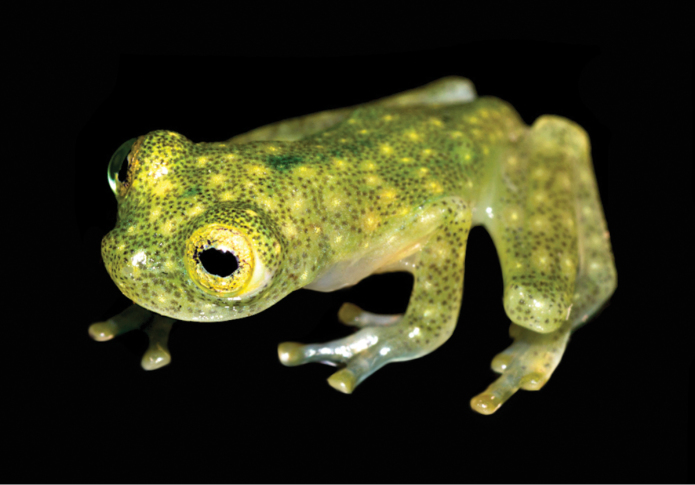
Juvenile of *Hyalinobatrachium yaku* in life, QCAZ 53354.

The other male from the type locality (MZUTI 5002) has more foot webbing (I 1 — 2- II 0^+^ — 2^+^ III 1 — 2^1/3^ IV 2^1/3^ — 1 V) than the holotype. Juveniles have the same color pattern as adults, but the number and extent of the middorsal green dots varies, but they are usually smaller and less pronounced posteriorly (Fig. 4).

##### Vocalizations.

**Figure 5.**
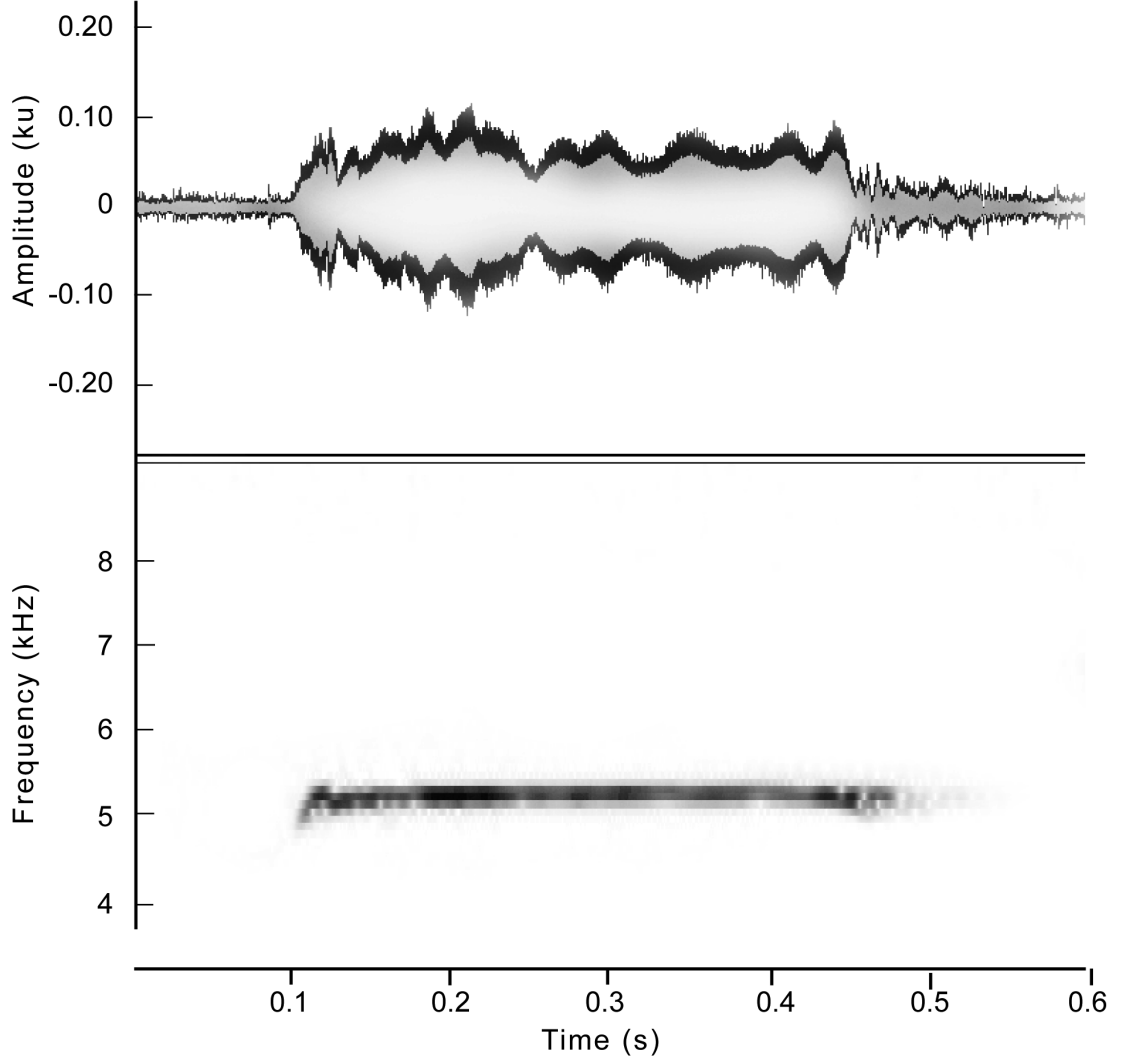
Call of *Hyalinobatrachium yaku* sp. n., holotype.

The description is based on a series of ten calls emitted by the holotype and recorded by JC. The advertisement call of *Hyalinobatrachium yaku* is a single and high pitched tonal note (Fig. 5). Neither frequency nor amplitude modulation was observed. The call lasts 0.27–0.4 s (0.3 ± 0.03) and has an average call rate of 9.0 calls/minute. Time between calls varied from 5.3–8.9 s (7.1 ± 1.1). The dominant frequency, which is included in the fundamental frequency, ranges from 5219.3–5329.6 Hz (5283.8 ± 35.0). The frequency band has a lower frequency of 5207.3–5314.8 Hz (5264.6 ± 34.6) and an upper frequency of 5236.5–5340.5 Hz (5299.1 ± 34.1).

##### Ecology.

At Kallana, the holotype and one paratype (MZUTI 5002) were found calling from the underside of leaves of riverine vegetation in pristine forest. The holotype was on the same leaf as two egg clutches, approximately 3 m above the stream. The paratype was also calling from the underside of a leaf nearly 6 m above water. The stream itself was slow-flowing, relatively narrow (approximately 3 m wide), and with depths no greater than 100 cm. Syntopic species at Kallana are: *Nymphargus mariae*, *Teratohyla midas*, *Agalychnis hulli*, *Phyllomedusa tomopterna*, *Hypsiboas calcaratus*, *H. geographicus*, *Osteocephalus fuscifacies*, *Pristimantis enigmaticus*, and *P. peruvianus*.

At Ahuano, the single individual was found on the underside of a leaf at 1 m above water in riverine vegetation along a small stream, tributary of the Arajuno River. The stream was slow-flowing, very narrow (approximately 1m wide), and shallow (approximately 40 cm deep). The area was covered by secondary forests. At Ahuano, *Hyalinobatrachium yaku* was found in syntopy with *Teratohyla midas* and *H. ruedai* (Cisneros-Heredia and McDiarmid 2006b).

Unlike individuals found at Kallana and Ahuano, individuals from San José de Payamino were found perched on leaves of small shrubs, ferns, and grasses (30–150 cm above ground) in disturbed secondary forest. All but one individual were found within a relatively small area near the Timburi Cocha Research Station bordering the Payamino River, with the additional individual found in slightly more mature secondary growth 50 m east of a dirt road situated approximately 1.5 km west of the research station (see Maynard et al. 2016). Additionally, all individuals recorded at San José de Payamino were found >30 m from any stream. Due to this unusual circumstance, syntopic species associated with *H. yaku* at San José de Payamino is rather extensive, as amphibian diversity in secondary forest at this site is high (Maynard et al. 2016). Syntopic glassfrog species include: *Cochranella resplendens*, *Hyalinobatrachium munozorum*, and *Teratohyla midas*. Other sympatric amphibian species include: *Allobates femoralis* (complex), *Hyloxalus sauli*, *Rhaebo ecuadorensis*, *Rhinella margaritifera*, *R. marina*, *Dendropsophus marmoratus*, *Hypsiboas boans*, *H. cinerascens*, *H. geographicus*, *H. punctatus*, *Nyctimantis rugiceps*, *Osteocephalus buckleyi*, *O. mutabor*, *Osteocephalus* sp., *Phyllomedusa tarsius*, *P. vaillantii*, *Scinax garbei*, *S. ruber*, *Hypodactylus nigrovittatus*, *Pristimantis acuminatus*, *P. altamazonicus*, *P. conspicillatus*, *P. croceoinguinis*, *P. delius*, *P. diadematus*, *P. kichwarum*, *P. lanthanites*, *P. librarius*, *P. variabilis*, P. aff. martiae, *Adenomera andreae*, *Engystomops petersi*, *Leptodactylus wagneri*, *Lithodytes lineatus*, *Chiasmocleis bassleri*, *Bolitoglossa peruviana*.

##### Distribution.

**Figure 6.**
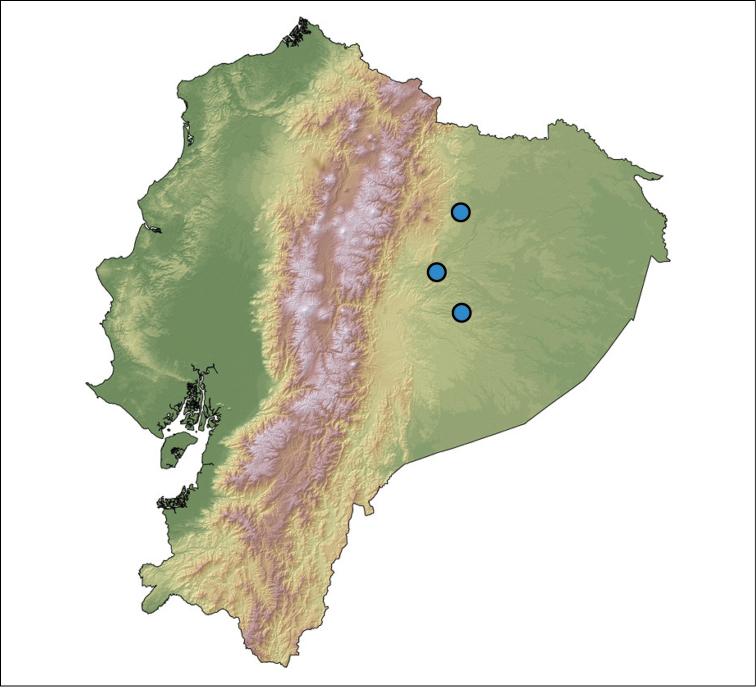
Distribution of *Hyalinobatrachium yaku* in Ecuador.

*Hyalinobatrachium yaku* is only known from three localities on the Amazonian lowlands of Ecuador at elevations between 300–360 m. The two most-distant sites, Kallana in province of Pastaza, and San José de Payamino in province of Orellana, are approximately 110 km from one another, while Ahuano, province of Napo, is midway between them (Fig. 6). Given the geographic distance between the localities where the new species has been found, it is likely that *H. yaku* has a broader distribution, including areas in nearby Peru.

##### Evolutionary relationships.

All inferred phylogenetic trees show that *Hyalinobatrachium yaku* and *H. pellucidum* are sister species (Fig. 2). Trees obtained for each mitochondrial gene trees are congruent with the tree shown in Figure 2.

##### Etymology.

The specific epithet *yaku* is the Kichwa word for *water*. Water, in the form of streams, is fundamental for the reproductive biology of all glassfrogs. Water pollution, mainly through oil and mining activities, represents one of the biggest threats for Amazonian amphibians, as well as for numerous other water-dependent species.

##### Conservation status.

Given that *Hyalinobatrachium* species are morphologically conserved and that many distinctive color traits are lost in preserved specimens (i.e., dorsal green spots), finding new records of *H. yaku* in herpetological collections is challenging. Also, many species of the genus are arboreal and difficult to find in nature, but this scarcity does not necessarily mean that the species have low abundances. Available information is insufficient to suggest an evaluation following IUCN criteria, thus we suggest that *H. yaku* is a Data Deficient species.

## Discussion

### Systematics

Although species delimitation within *Hyalinobatrachium* is often times complex (Castroviejo-Fisher et al. 2011a,b). The validity of *H. yaku* sp. n. is supported from all analyzed datasets (morphology, calls, DNA), allowing unambiguous separation from all congenerics, including its most related taxon, *H. pellucidum*. Our study also shows the importance of having a good record of coloration in life, especially in groups like glassfrogs where intraspecific coloration is relatively low (with the notable exception of *Espadarana prosoblepon*; see Savage 2002; Cisneros-Heredia and McDiarmid 2007; Arteaga et al. 2013). As mentioned above, the characteristic dark green spots present on the head and dorsum of *H. yaku* leave no trace in preserved specimens; thus, taxonomic work where only museum material is available may result in an underestimation of diversity. However, careful examination of some morphological and chromatic pattern together with the provenance of specimens could be useful to discriminate the identity of some specimens, in particular those in better preservation status. For example, even though *Hyalinobatrachium yaku*, *H. pellucidum* and *H. anachoretus* are basically indistinguishable in preservation, when dorsal coloration has faded, identification of specimens is still possible because, as far as we know, the species are allopatric. While *H. yaku* occurs in the Lowland Amazonian forests of Ecuador below 400 m, *H. anachoretus* is only known from Cloud forests at Abra Patricia, northeastern Peru at 2001 m (Twomey et al. 2014), and *H. pellucidum* inhabits the Foothill, Low Montane, and Cloud forests on the eastern versant of Cordillera Oriental of the Andes of Ecuador and Peru, above 1300 m in northeastern Ecuador and above 1000 m in southeastern Ecuador and eastern Peru (although there are two localities down to 500 m in the Tarapoto area, northeastern Peru) and up to 1740 m (Lynch and Duellman 1973; Duellman and Schulte 1993; Cisneros-Heredia and McDiarmid 2006a,b, 2007; Castroviejo-Fisher et al. 2009; Twomey et al. 2014; cited as *Hyalinobatrachium* sp. from the Chinchipe River, SW Cordillera del Condor by Cisneros-Heredia et al. 2008). *Hyalinobatrachium yaku* is sympatric with *H. munozorum* and *H. ruedai*, but they are distinguished by characters that are fairly evident even in preserved specimens (pericardium coloration, dorsal melanophores pattern and snout form; see Diagnosis).

The inferred phylogeny confirms some pending taxonomic issues within *Hyalinobatrachium*; for example, only based on molecular data, there are at least four unconfirmed candidate species (see Murray and Schleifer 1994; Padial et al. 2010): *Hyalinobatrachium* sp. (MIZA 317) from Venezuela; *Hyalinobatrachium* sp. (MAR 2147, 2222) from Colombia; Hyalinobatrachium aff. bergeri (MTD 46305, MHNC 5577) from Peru; and a cryptic species within *H. colymbiphyllum* (Fig. 2). Also, it is very likely that more glassfrog species are yet to be found not just in unexplored areas of the Amazon basin, but also in rather well known areas, glassfrogs become highly arboreal and are difficult to find outside of the breeding season (e.g, Señaris and Ayarzaguena 2005; Castroviejo-Fisher et al. 2009; Twomey et al. 2014). Additionally, the revision of taxa with large and/or discontinuous distributions will certainly reveal cryptic diversity (see Castroviejo-Fisher et al. 2011b; Gehara et al. 2014).

### Conservation

Although the Amazon basin is globally recognized by its incredible biological and cultural diversity (Bass et al. 2010, Tarazona-Santos et al. 2001), current and future threats to conservation are conspicuous. For example, even though a high proportion of the Ecuadorian Amazon is already concessioned to several extractive activities (see Lessmann et al. 2016), the Government of Ecuador is planning to intensify oil extraction in the region (e.g., Ishpingo-Tambococha-Tiputini project, XI Ronda Petrolera). Aside from obvious concerns such as water pollution, extraction of natural resources increases the level of regional road development, which could threaten populations of *H. yaku* due to habitat degradation and isolation.

At San José de Payamino, the presence of a dirt road has been shown to negatively influence amphibian abundance and diversity, and alter assemblage composition (Maynard et al. 2016). All records of *H. yaku* at this site were > 1 km away from the road edge. Glassfrogs presumably require continuous tracts of forest to interact with nearby populations, and roads potentially act as barriers to dispersal for transient individuals, such as those documented at San José de Payamino.

Considering the current scenario of development in the Ecuadorian Amazon, alternatives that contemplate both conservation and different levels of exploitation have been put forward by the scientific community (see Lessmann et al. 2016). These alternatives need to be seriously considered, especially when biodiversity research and conservation are clearly identified, at least in theory, as priorities for the Ecuadorian Government (Plan Nacional del Buen Vivir 2013–2017).

## Author contributions

Manuscript writing was led by JMG, with substantial contributions by all authors. Ecological data were obtained by RJM, PSH, JC, RLL and DFCH. Molecular data were analyzed by JMG. Morphological descriptions, measurements, and species comparisons were made by JMG and DFCH.

## Supplementary Material

XML Treatment for Hyalinobatrachium yaku

## Appendix 1

**Examined specimens**

*Hyalinobatrachium esmeralda*: Colombia: Departamento de Boyacá, Municipio de Pajarito, Inspección Policía Corinto, finca ‘El Descanso’, quebrada ‘La Limonita’, 1600-1650 m, ICN 9592–94, 9596, 9602–03 (type series of *H. esmeralda*).

*Hyalinobatrachium pellucidum*: Ecuador: *Morona Santiago*: Nueva Alianza, Finca Santa Catalina (78.1335°W, 2.100°S, 1305 m), Límite del Parque Nacional Sangay, MEPN 14706. Quebrada del Río Napinaza (78.4070°W, 2.9266°S, 1100 m, QCAZ 42000. *Sucumbíos*: Río Azuela (0.1167 S, 77.6167 W; 1740 m), Quito–Lago Agrio road; KU 164691 (holotype), USNM 286708–10; Río Reventador, USNM 286711–12. *Morona Santiago*: km 6.6 on the Limón-Macas road (ca. 2.92816S, 78.344W; 1013 m), QCAZ 29438; 6 km N of Limon, QCAZ 25950. *Provincia de Zamora Chinchipe*: Cordillera del Cóndor, Miazi Alto (4.25044 S, 78.61356 W; 1282 m), QCAZ 41560–61, 41648.

*Hyalinobatrachium munozorum*: Ecuador: *Provincia de Sucumbíos*: Santa Cecilia (00°03'N, 76°58'W; 340 m), KU 118054 (holotype), 105251, 123225, 150620 (paratypes), 152488–89, 155493–96, 175504. *Provincia de Orellana*: Tiputini Biodiversity Station, ZSFQ DFCH-USFQ D105. Colombia: Departamento del Meta: Meta, ICN 5031-34, 39503. *Departamento de Amazonas*: Leticia, ICN (serie de campo JMR 4119).

*Hyalinobatrachium ruedai*: Colombia: *Departamento de Caquetá*: Parque Nacional Natural de Chiribiquete, ICN 40409 (holotype), ICN 40410-11, IND-AN 5448-52 (paratypes). Ecuador: *Provincia de Napo*: Tena, ZUSF DFCH-USFQ 0735. *Provincia de Pastaza*: Río Manderoyacu, Arajuno, MEPN 6427.
